# Histone chaperone Nap1 dismantles an H2A/H2B dimer from a partially unwrapped nucleosome

**DOI:** 10.1093/nar/gkad396

**Published:** 2023-05-13

**Authors:** Fritz Nagae, Shoji Takada, Tsuyoshi Terakawa

**Affiliations:** Department of Biophysics, Graduate School of Science, Kyoto University, Kyoto, Japan; Department of Biophysics, Graduate School of Science, Kyoto University, Kyoto, Japan; Department of Biophysics, Graduate School of Science, Kyoto University, Kyoto, Japan; PREST, Japan Science and Technology Agency (JST), Kawaguchi, Japan

## Abstract

DNA translocases, such as RNA polymerases, inevitably collide with nucleosomes on eukaryotic chromatin. Upon these collisions, histone chaperones are suggested to facilitate nucleosome disassembly and re-assembly. In this study, by performing *in vitro* transcription assays and molecular simulations, we found that partial unwrapping of a nucleosome by an RNA polymerase dramatically facilitates an H2A/H2B dimer dismantling from the nucleosome by Nucleosome Assembly Protein 1 (Nap1). Furthermore, the results uncovered molecular mechanisms of Nap1 functions in which the highly acidic C-terminal flexible tails of Nap1 contribute to the H2A/H2B binding by associating with the binding interface buried and not accessible to Nap1 globular domains, supporting the penetrating fuzzy binding mechanism seemingly shared across various histone chaperones. These findings have broad implications for the mechanisms by which histone chaperones process nucleosomes upon collisions with translocases in transcription, histone recycling and nucleosomal DNA repair.

## Graphical Abstract

**Graphical Abstract:**
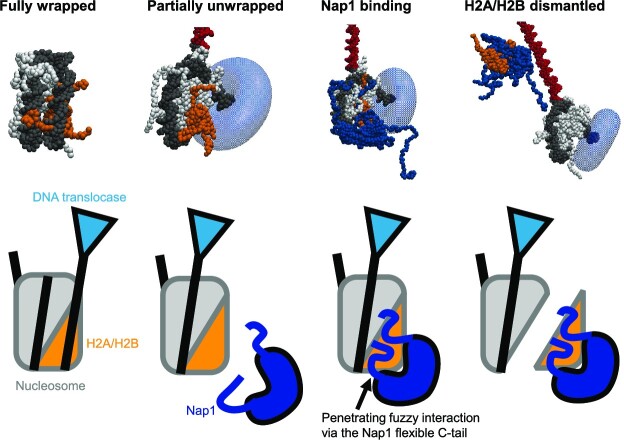

## INTRODUCTION

Nucleosomes are repeating structural units of eukaryotic chromatin, composed of two H2A/H2B dimers and one H3/H4 tetramer, wrapped by 147 bp DNA (1). Since the wrapped nucleosomal DNA is largely occluded, nucleosomes serve as general repressing elements. Thus, their genomic positioning has regulatory functions and is controlled by multiple mechanisms (2,3). Nucleosomes are also an essential platform for carrying epigenetic information. On chromatin, various DNA translocases, such as RNA polymerases (RNAPs), replicative helicases and exonucleases, move unidirectionally along DNA and inevitably collide with the nucleosomes. Nucleosomes must be appropriately processed upon the collisions to maintain their genomic positions and epigenetic information (2,3). However, the molecular mechanisms of nucleosome processing remain unclear.

A previous *in vitro* study suggested that the collision between a model translocase, T7 RNAP, and a nucleosome induces downstream nucleosome repositioning by the lane-switch mechanism (4). In this mechanism, after a translocase partially unwraps nucleosomal DNA up to –18 bp from the dyad, the remaining wrapped DNA switches its binding lane to that vacated by the unwrapping. Subsequently, the downstream DNA rewraps, completing the downstream repositioning. Notably, the study used the RNAP from bacteriophage T7, which should not have any evolutionarily selected interactions with nucleosomes. Thus, the downstream repositioning of nucleosomes via the lane-switch mechanism should be a generic outcome of the translocase collision. On the other hand, the downstream repositioning severely perturbs the genomic positioning of nucleosomes and thus is not desired for genomic sustainability. Therefore, we expect that there must be some evolutionarily gained mechanisms that prevent such downstream repositioning. Furthermore, since the lane-switch occurs around –18 bp from the dyad, nucleosomes should be appropriately processed before that point to maintain the chromatin state, which is required for transcription through nucleosomes or histone recycling.

Histone chaperones, which can bind to histones in a nucleosome, are one of the promising candidate proteins to process a nucleosome upon collision (5). Among many, Nucleosome Assembly Protein 1 (Nap1) is a small histone chaperone with a molecular weight of 48 kDa as a monomer and stably forms a homodimer in solution (6). A Nap1 homodimer (simply Nap1, hereafter) is known to bind to a single H2A/H2B dimer with a nanomolar range dissociation constant (7,8) and to promote nucleosome assembly (9). A previous study showed that Nap1 slowly dismantles H2A/H2B dimers from a fully wrapped nucleosome at a low temperature (4°C) on a time scale of hours (10). Other studies suggested that a positively coiling stress (11) on DNA or the RSC chromatin remodeling complex (12) facilitates dismantling of the H2A/H2B dimer or histone octamer in the presence of Nap1 potentially by loosening nucleosomal DNA wrapping. These reactions proceed on a time scale of minutes. The recently solved cryogenic electron microscopic structure showed that an H2A/H2B dimer dissociates from a nucleosome partially unwrapped by eukaryotic RNA polymerase II in the presence (13) or absence (14) of histone chaperones. However, whether a partial DNA unwrapping by a translocase facilitates the H2A/H2B dismantling by Nap1 in a physiological-like setting has not been investigated, and the molecular mechanism of the facilitation remains unknown.

Nap1 has flexible tails on its N- and C-termini, which play pivotal roles in its function (8,10,15). In particular, the intrinsically disordered C-terminal tails are required to slowly dismantle H2A/H2B dimers from a nucleosome (10), although they are dispensable for binding to an H2A/H2B dimer in solution (8,15). However, the flexibility of this region has prevented us from relating their structural property and function. For example, this region was truncated in the previous crystallographic study of the complex of Nap1 and an H2A/H2B dimer (16).

In this study, we performed *in vitro* transcription assays with nucleosome substrates, followed by electrophoresis mobility shift assays (EMSAs), preparative gel electrophoresis assays and molecular dynamics simulations, demonstrating that partial unwrapping of a nucleosome by a polymerase dramatically facilitates an H2A/H2B dimer dismantling from the nucleosome by Nap1 and that Nap1 C-terminal flexible tails play a crucial role in the dismantling. We used T7 RNAP as a model translocase in the *in vitro* transcription assays to isolate the effect of Nap1 on the dismantling by eliminating possible evolutionarily selected interactions between helicases and nucleosomes. In the simulations, which provided unprecedented details of molecular dynamics, especially in the presence of flexible protein regions, we also observed that Nap1 binds to and dismantles an H2A/H2B dimer from a partially unwrapped nucleosome, consistent with the experimental results. The highly acidic C-terminal flexible tails of Nap1 contributed to the binding by associating with the binding interface of H2A/H2B buried and not accessible to the Nap1 globular domains, suggesting the penetrating fuzzy binding mechanism seemingly shared across various histone chaperones. In the fuzzy binding (17), the Nap1 C-terminal tails have neither a stable structure, even when it binds to an H2A/H2B dimer, nor specific residues to interact with it. The findings may have broad implications for the mechanisms by which histone chaperones process nucleosomes upon collisions with translocases in transcription, histone recycling and nucleosomal DNA repair.

## MATERIALS AND METHODS

### Material preparations

DNA substrates for nucleosome reconstitution (Supplementary Table S1) were chemically synthesized (Eurofins Genomics) and amplified by polymerase chain reaction (PCR). We purified budding yeast histones (H2A, H2B, H3, H4, H3 V35C and H2B T119C), and Nap1, His^6^-Nap1 and Nap1ΔC (1–365) from an *Escherichia coli* protein expression system as previously described (6,18,19) (Supplementary Figure S1A, B). H3 V35C and H2B T119C were labeled with Alexa Fluor 647 C2 Maleimide and Alexa Fluor 488 C5 Maleimide (A20347 and A10254; Thermo Fisher Scientific) according to the manufacturer’s instructions. Also, we reconstituted nucleosomes using these materials by salt dialysis (18) (Supplementary Figure S1C).

### Electrophoresis mobility shift assays

We performed the *in vitro* transcription assays using the reconstituted nucleosomes in the presence and absence of Nap1 employing the HiScribe T7 High Yield RNA synthesis kit (New England BioLabs; E2040S) by following the manufacturer's instructions. In this assay, T7 RNAP transcribes the DNA substrate from the T7 promoter sequence next to the modified Widom 601 nucleosome positioning sequence. We exchanged adenine and thymine in some base pairs in the original 601 sequence so that T7 RNAP, for the first time, encounters the stalling thymine nucleotide –14 bp away from the dyad (Supplementary Table S1). The reactions containing 0 or 6 μM Nap1 dimers, 0.2 μM DNA substrates, 0.8 μM T7 RNAP, 1 mM GTP, 1 mM CTP, 1 mM UTP and 200 mM NaCl were incubated at 37°C for 10 min. The reaction products were run on a 6% TBE-polyacrylamide gel at 10.5 mA and 4°C for 2.5 h for separation according to electrophoretic mobilities. The gels were stained with SYBR Gold Nucleic Acid Gel Stain (Thermo Fisher Scientific; S11494), imaged using the iBright FL 1500 Imaging System (Thermo Fisher Scientific) and analyzed using Image J software (20).

### Preparative gel electrophoresis assays

We performed the preparative gel electrophoresis assays to identify the components of each band obtained in the EMSAs. In these assays, the 100 μl reactions containing 6 μM His^6^-Nap1 dimers, 0.2 μM nucleosomes, 0.8 μM T7 RNAP, 1 mM GTP, 1 mM CTP, 1 mM UTP and 200 mM NaCl were incubated at 37°C for 10 min. Then, we added the same volume of Ni-NTA agarose to the reactions, rotated the mixtures at 4°C for 30 min to pull down His^6^-Nap1 and fractionated the reaction products with the Prepcell apparatus (Bio-Rad; 1702980). Each fraction was run on 4% TBE- and 5–20% sodium dodecylsulfate (SDS)-polyacrylamide gels with a constant current of 10.5 and 21.0 mA applied at 4 and 25°C for 90 and 75 min, respectively. The native and denatured gels were stained with SYBR Gold Nucleic Acid Gel Stain and Ez Stain Silver (ATTO; AE-1360), respectively, imaged using the iBright FL 1500 Imaging System and analyzed using Image J software (20).

### Micrococcal nuclease assays

The reaction products were further analyzed by micrococcal nuclease (MNase) assays. First, the reaction buffer was exchanged to the CutSmart buffer (New England BioLabs; B7204) using a 30 K MWCO Amicon Ultra-0.5 Centrifugal Filter (Merck; UFC 503096). Then, we added 0.4 μl of 20 U/μl MNase (New England BioLabs; M0247S) to the reaction products and incubated them at room temperature for 30 and 60 min. The DNA fragments were purified using a spin column (Promega; A9281) and were run on e-PAGEL (ATTO; 2331830) in Tris-glycine buffer at 21 mA and 25°C for 75 min. The gels were stained with SYBR Gold Nucleic Acid Gel Stain, imaged using the iBright FL 1500 Imaging System and analyzed using Image J software (20).

### Coarse-grained molecular dynamics simulations

We used the same coarse-grained models as the previous study, where the collision between a translocase and a nucleosome was investigated (4). Briefly, we used the AICG2+ model for proteins [please refer to the original work (21) for details]. In this model, each amino acid was represented by one bead located on the Cα atom position, and native structure-based contact potentials stabilized the native structures. The dynamics of disordered regions, in which stable native structure is not available, were dictated by local potentials derived from the statistical analysis of the loop regions in the protein data bank structures (22). On the other hand, we used the 3SPN.2C model for DNA [please refer to the original work (23) for details]. In this model, each nucleotide was represented by three beads located at the positions of base, sugar and phosphate units, and the potentials stabilized the B-form structure. Electrostatic and excluded volume interactions were applied for interactions between proteins and DNA. Hydrogen bonding interactions were also applied for interactions between histones and DNA (24). The RESPAC algorithm decided a charge value on each surface bead so that the electrostatic potential around the protein reproduced that calculated from the all-atom model (25). An integer charge was put on each charged residue bead in disordered regions and each phosphate bead in DNA in its interaction with proteins. Only excluded volume interactions were applied for interactions between a torus-shaped translocase and the rest of the system.

We used the Langevin dynamics with a step size of 0.3 in the CafeMol time unit for the time propagation. The temperature and friction constant were set to 300 K and 0.843, respectively. The dielectric constant was set to 78.0, and the monovalent ion concentration was set to 300 mM unless otherwise specified. In this condition, the simulations in the previous study (26) reproduced the experimentally suggested metastable state in a nucleosome assembly and the equilibrium constant of the assembly reaction (27). We performed all the simulations using CafeMol 3.2 (https://www.cafemol.org) (28).

The initial structure of a nucleosome was prepared using coordinates in the crystal structures of a histone octamer (PDB ID: 1KX5) (29) and the Widom 601 DNA sequence (PDB ID: 3LZ0) (30). First, 32 and 92 bp double-stranded DNA segments were connected to the nucleosome as the upstream and downstream sequences, respectively. Then, the upstream DNA was threaded through the translocase and pulled so that the translocase collided with the nucleosome. After the translocase unwraps nucleosomal DNA up to –31 bp away from the dyad, we added (unless otherwise specified) a Nap1 molecule, the coordinates of which were from the crystal structure (PDB ID: 2Z2R) (31) for the globular domains (residues 74–365) and PyMOL builder (https://pymol.org) for the disordered regions (residues 1–73 and 366–417). We put it 80 Å away from the nucleosome to prepare the initial structures. To focus on the mechanistic role of Nap1, the simulations were performed with 50 nM Nap1, which is more dilute than the estimated cellular concentration (μM order) (6). In each simulation, the base pair –31 bp away from the dyad was anchored to the center of the translocase. First, by performing the 1 × 10^6^ step molecular dynamics simulation, we obtained the structure in which Nap1 bound to a partially unwrapped nucleosome. Starting from this structure, we conducted 3 × 10^8^ step production simulations, recording snapshot structures at every 1 × 10^4^ steps. As controls, we also performed the simulations where a fully wrapped nucleosome with and without Nap1 and a partially unwrapped nucleosome without Nap1 are in each initial structure for 3 × 10^8^ steps.

### Binding free energy calculations

Using the coarse-grained models, we performed umbrella sampling simulations to calculate free energy values against the distance between the centers of mass (COM) of Nap1 (or Nap1ΔC) and H2A/H2B globular domains. In each of the 44 setups, a harmonic umbrella potential [spring coefficient *k* is set to 1.0 kcal/(mol Å^2^)] was applied to restrain the distance to 25 + 2*i*Å (*i* = 0, 1, 2 ⋯ 43). The coordinates of five replicates of the 44 setups were updated for 1 × 10^8^ steps. The free energy profile was reconstructed using the WHAM algorithm from these simulation trajectories (32).

## RESULTS

### Nap1 induces H2A/H2B dismantling from a partially unwrapped nucleosome

**Figure 1.**
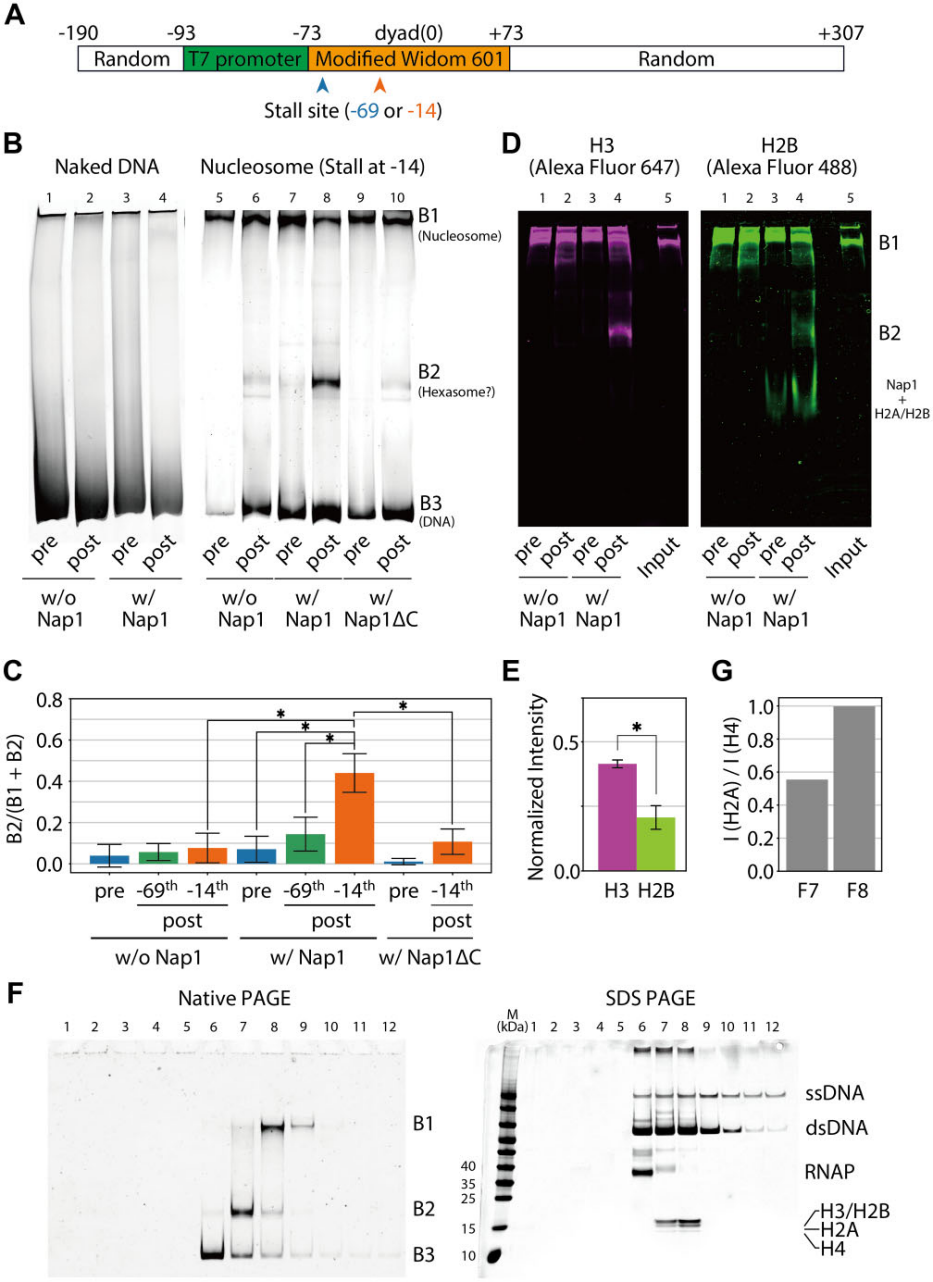
*In vitro* transcription assays. (**A**) The DNA sequence in the assay. The blue and orange arrows point to the T7 RNAP stall sites. (**B**) Gel images of native PAGE of the reaction products using naked DNA (lanes 1–4) or DNA reconstituted with a nucleosome (lanes 5–10) as a substrate in the presence or absence of Nap1 or C-terminal tail-truncated Nap1 (Nap1ΔC). ‘pre’ and ‘post’ refer to pre- and post-initiation of transcription, respectively. In the reactions, ATP was omitted to make T7 RNAP stall at the sites depicted in (A). (**C**) The intensity of B2 normalized by the total intensity of B1 and B2. The bars and error bars represent the mean and standard deviation (SD) from three replicates. The asterisks represent *P* < 0.05 by Student's *t*-test. (**D**) Gel images of native PAGE of the reaction products using DNA reconstituted with a nucleosome in the presence of T7 RNAP and the presence or absence of Nap1. The nucleosome was reconstituted with Alexa Fluor 647-labeled H3, Alexa Fluor 488-labeled H2B, H4 and H2A. The same gel was imaged using the filters for Alexa Fluor 647 (left) and Alexa Fluor 488 (right), respectively. ‘Input’ refers to the reconstituted nucleosome without T7 RNAP and Nap1. (**E**) The intensities of Alexa Fluor 647 (H3) and Alexa Fluor 488 (H2B) in the B2 band [lane 4 in (D)] are normalized by the intensities of Alexa Fluor 647 and Alexa Fluor 488 in the band of input nucleosomes [lane 5 in (D)], respectively. The bars and error bars represent the mean and SD from three replicates. The asterisks represent *P* < 0.05 by Student's *t*-test. (**F**) Gel images of native PAGE (left) and SDS–PAGE (right) of the fractions obtained by the preparative gel electrophoresis. ‘M’ stands for a marker (Thermo Fisher Scientific; 26617). (**G**) The intensity ratio of H2A to H4 of F7 in (F right) normalized by that of F8.

To reveal the effects of Nap1 on the structural modulation of a partially unwrapped nucleosome, we added Nap1 molecules to nucleosomes partially unwrapped by T7 RNAPs in *in vitro* transcription assays. The budding yeast Nap1 and the histones were purified from *E. coli* as previously described (6) (see the Materials and Methods for details). Nucleosomes were reconstituted with the purified histones and 498 bp linear DNA substrates containing the T7 promoter and the modified Widom 601 nucleosome positioning sequence (Figure 1A). The Widom 601 sequence was modified so that adenines in the template strand were exchanged to thymines from the entry (–73 bp from the dyad) to the stalling (–14 bp from the dyad) nucleotide (Supplementary Table S1). In this setup, T7 RNAPs, which begin translocation from the T7 promoter sequence, unwrap ∼59 bp nucleosomal DNA and stall at the stalling nucleotide in the presence of UTP, CTP and GTP, and in the absence of ATP (4). Our previous study validated the RNAP stalling [see supplementary figure S9 in (4)]. A previous study has shown that T7 RNAP transcription is moderately inhibited when a DNA substrate reconstituted with four or seven nucleosomes is transcribed (33). However, significant inhibition was not observed when a DNA substrate reconstituted with a single nucleosome was transcribed by adding all four nucleotides (Supplementary Figure S2). Therefore, the effect of nucleosomes on interfering with T7 RNAP is likely to be weak.

After 10 min of incubation, we stopped the reactions by adding EDTA and performed native polyacrylamide gel electrophoresis (PAGE) of the products. As a result, we observed three distinct bands on the gel [B1, B2 and B3 in Figure 1B (lane 8); ‘pre’ and ‘post’ refers to pre- and post-initiation of transcription, respectively], though almost no B2 band was detected before transcription was initiated by adding UTP, CTP and GTP (lane 7). To identify the molecular components in these bands, we performed the control experiments in which T7 RNAP was added to naked DNA. In these experiments, only B3 was observed regardless of the presence of Nap1 or UTP, CTP and GTP, suggesting that B3 contains DNA and possibly T7 RNAP (lanes 1–4). We also performed the control experiments in which T7 RNAP was added to nucleosomes in the absence of Nap1. In these experiments, strong intensities of only B1 and B3 were observed, suggesting that B1 contains nucleosomes and possibly T7 RNAP (lanes 5 and 6). Notably, strong intensities of only B1 and B3 were detected when Nap1 molecules were added to nucleosomes without transcription, indicating that the Nap1 binding to a nucleosome is transient, if indeed it occurs. When we used DNA substrates with the sequence designed to stall T7 RNAP at –69 bp from the dyad instead of –14 bp (Supplementary Table S1), we did not observe significant amounts of B2 regardless of the presence of Nap1 (Figure 1C). This result supported that B2 production requires a certain degree (at least >4 bp) of partial unwrapping of nucleosomal DNA.

To identify the molecular components in B2, we sought to repeat the same experiment with fluorescent histones. To achieve this, first, mutants in which V35 of H3 and T119 of H2B were replaced with cysteines were purified from *E. coli* and were conjugated with Alexa Fluor 647 C_2_ Maleimide and Alexa Fluor 488 C_5_ Maleimide (Thermo Fisher Scientific; A20347 and A10254). Then, we reconstituted nucleosomes using wild-type H4 and H2A and these labeled mutants. Following this, the nucleosomes were partially unwrapped by T7 RNAP and were subjected to native PAGE. As a result, both Alexa Fluor 647 and Alexa Fluor 488 signals could be observed at the B2 band position (Figure 1D; Supplementary Figure S3). In addition, the signal intensities were measured and normalized with the band intensities of the input nucleosomes (lane 5 in Figure 1D), respectively. As a result, the intensity of Alexa Fluor 647 (H3) was found to be ∼2.0 times that of Alexa Fluor 488 (H2B) (Figure 1E). These results supported that B2 is a band derived from hexasomes. We also found the complex of H2B (or H2A/H2B) and Nap1 in B3.

To further confirm that B2 is a band derived from hexasomes, we performed preparative gel electrophoresis of the reaction in which Nap1 molecules were added to partially unwrapped nucleosomes (Figure 1F). The preparative gel electrophoresis can fractionate molecular components in a reaction product by size. After the fractionation, each fraction was run on native (Figure 1F left) and denaturing (Figure 1F right) gels to reveal the components. The native PAGE demonstrated that we successfully obtained the fractions F6 containing B3 (99%), F7 containing B2 (63%) and F8 containing B1 (76%) (Figure 1F left). Here, quantifying the amounts of each histone (H3, H4, H2A or H2B) in F7 can identify the molecular components in B2. Indeed, the SDS–PAGE of F7 showed that B2 contains all the histone species (Figure 1F right). The band intensities were analyzed, and the normalized ratio of H2A to H4 was ∼1:2 when we assume that F8 mainly contains a nucleosome and that the ratio is 1:1 (Figure 1G). Interestingly, this result suggests that the molecular components in F7 mainly contain hexasomes. F7 also includes RNAP, supporting that hexasomes are produced via partial unwrapping caused by RNAP. In summary, the preparative gel electrophoresis assay supported that B2 is a band derived from hexasomes.

The results of the EMSAs (Figure 1B–E) and the preparative gel electrophoresis assay (Figure 1F, G) suggested that adding Nap1 to a nucleosome partially unwrapped by T7 RNAP induces H2A/H2B dismantling and hence produces a hexasome. To further confirm this, we performed MNase assays (Supplementary Figure S4A). When the fully wrapped or partially unwrapped nucleosomes in the presence and absence of Nap1 were digested by MNase, we observed the ∼156 bp bands. However, when the partially unwrapped nucleosomes in the presence of Nap1 were digested, we observed the ∼120 bp band and the ∼156 bp band. The lengths of ∼156 and ∼120 bp are consistent with those of the digestion products of nucleosomes (34) and hexasomes (35), respectively. Although a significant amount of the partially unwrapped nucleosomes were processed to hexasomes in the presence of Nap1, the substrates still contain a considerable amount of fully wrapped nucleosomes (4), explaining the ∼156 bp bands. Even when the MNase reaction time was increased from 30 to 60 min, the intensity of the ∼120 bp bands of the partially unwrapped nucleosome in the presence of Nap1 is moderately greater than its absence, suggesting that this result is robust to the MNase reaction time (Supplementary Figure S4B). Therefore, the MNase assays also supported that Nap1 induces H2A/H2B dismantling from a partially unwrapped nucleosome.

As mentioned above, the previous study showed that Nap1 C-terminal tails (residues 366–417), but not the N-terminal tail, are required to slowly dismantle an H2A/H2B dimer from a nucleosome on a time scale of hours (10). To determine if the tails play roles in H2A/H2B dismantling from a partially unwrapped nucleosome, we performed native PAGE of the reaction products using C-terminal tail-truncated Nap1 (Nap1ΔC) (Figure 1B). As a result, we did not observe the significant intensity of the B2 bands (hexasomes) regardless of partial unwrapping (Figure 1C). This result showed that Nap1 C-terminal tails play a significant role in H2A/H2B dismantling from a partially unwrapped nucleosome.

### Simulation model validation: molecular dynamics simulations reproduced the binding interface between Nap1 and an H2A/H2B dimer

To investigate the detailed molecular dynamics by which Nap1 dismantles an H2A/H2B dimer from a partially unwrapped nucleosome, we sought to perform molecular dynamics simulations of Nap1 binding to a partially unwrapped nucleosome. Since molecular dynamics simulations require costly computational resources, it is daunting to simulate this system for a reasonable time scale with all the atoms explicitly treated. Previously, molecular dynamics simulations with our coarse-grained model successfully reproduced nucleosome repositioning upon a collision with a translocase via a partially unwrapped intermediate (4). In the coarse-grained model, one amino acid and one nucleotide were represented by one and three beads, respectively. Forces derived from the potential energy functions stabilized native protein and B-form double-stranded DNA structures (21,23). Also, the electrostatic and hydrogen-bonding interactions successfully reproduced the canonical nucleosome structure (24), and the Langevin dynamics with the implicit solvent treatment dramatically sped up the simulations (36).

Here, we modeled Nap1 and its interaction with an H2A/H2B dimer similarly. The potential energy functions stabilized the native Nap1 structure (PDB ID: 2Z2R) (31), and the statistics-based flexible local potentials modeled the N- and C-terminal tails (22). Again, only electrostatic and excluded volume interactions were considered as interactions between Nap1 and an H2A/H2B dimer.

**Figure 2.**
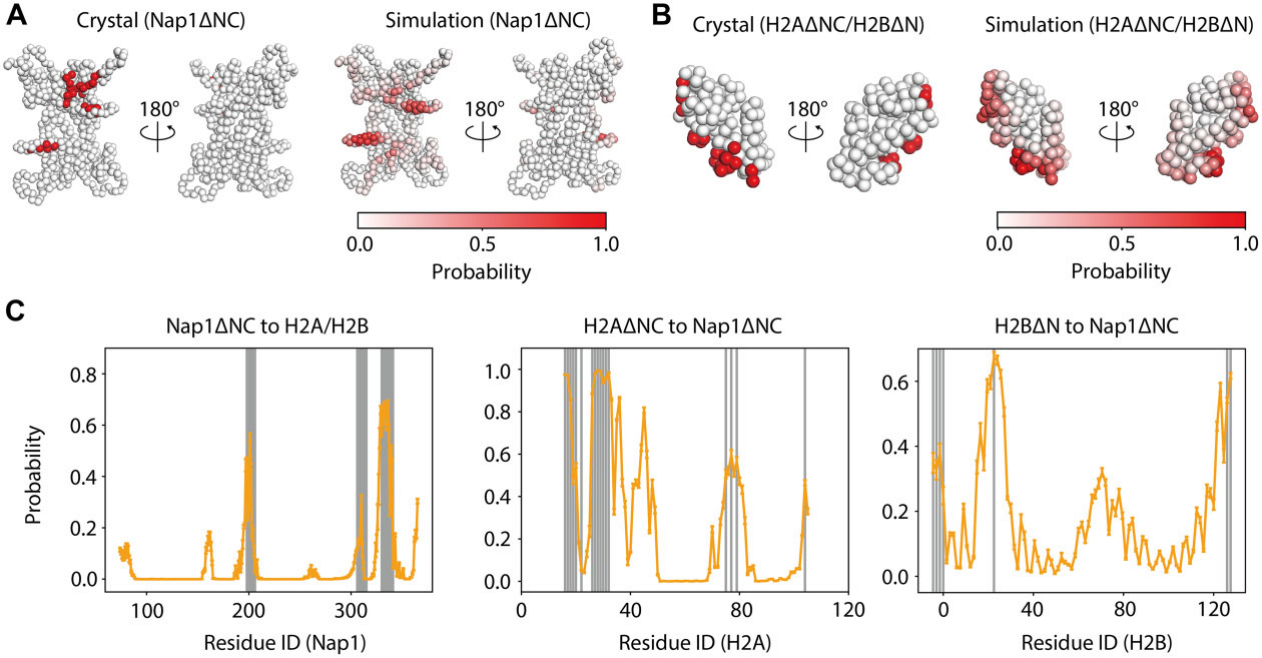
Validation of the coarse-grained model. (**A**) The structures of Nap1ΔNC (residues 74–365) in the one bead per one amino acid representation. The residues contacting the H2AΔNC (residues 16–105)/H2BΔN (residues 37–125) dimer in the crystal structure (PDB ID: 5G2E) are colored red in the left panel. The red gradient in the right panel shows the probabilities of each residue contacting the H2AΔNC/H2BΔN dimer in the simulations. Two coarse-grained residues with a van der Waals radius of 1.9–2.6Å were considered to be in contact with each other when they were within 10 Å. (**B**) The structures of the H2AΔNC/H2BΔN dimer in the one bead per one amino acid representation. The residues contacting Nap1ΔNC in the crystal structure (PDB ID: 5G2E) are colored red in the left panel. The red gradient in the right panel shows the probabilities of each residue contacting Nap1ΔNC in the simulations. (**C**) The probability of residues in Nap1ΔNC contacting H2AΔNC/H2BΔN (left), in H2AΔNC contacting Nap1ΔNC (center) and in H2BΔN contacting Nap1ΔNC (right). Gray vertical lines represent residues in contact in the crystal structure (PDB ID: 5G2E). Error bars represent SDs calculated from the 20 simulations.

It is not trivial whether this simple treatment can reproduce the complex conformation of Nap1 and H2A/H2B. Therefore, we performed coarse-grained molecular dynamics simulations of Nap1 and H2A/H2B to reproduce the structure. For structural validation, we used the crystal structure of Nap1 and H2A/H2B (16). In the crystal structure, the Nap1 N-terminus (residues 1–73) and C-terminus (residues 366–417), the H2A N-terminus (residues 1–15) and C-terminus (residues 106–128) and the H2B N-terminus (residues 1–36) were truncated. Therefore, we used the truncated construct in our simulations. It is known that the histone tails do not have a significant stabilizing effect on the Nap1 binding to H2A/H2B (8). From the simulation trajectories, we calculated the probabilities of each residue in Nap1ΔNC (H2AΔNC/H2BΔN) contacting those in H2AΔNC/H2BΔN (Nap1ΔNC) and compared them with the contacts in the crystal structure (Figure 2A, B). As a result, we found that the Nap1ΔNC residues 195–202, 304–310 and 321–351 frequently contact the H2AΔNC/H2BΔN residues as in the crystal structure (Figure 2C left). Also, the H2AΔNC residues 16–21, 24–38, 39–49 and 70–82, and the H2BΔN residues 37–40, 50–60, 80–93 and 118–125 frequently contact the Nap1ΔNC residues (Figure 2C center and right). Of them, the H2AΔNC residues 16–21, 24–38 and 70–82, and the H2BΔN residues 37–40, 50–60 and 118–125 also contact Nap1ΔNC residues in the crystal structure. On the other hand, the H2AΔNC residues 39–49 and the H2BΔN residues 80–93 do not contact the Nap1ΔNC residues in the crystal structure. Consistent with the simulations, however, the previous hydrogen–deuterium exchange assays showed that these residues are in contact with Nap1 residues (37) in solution. Therefore, these simulations suggested that our coarse-grained model successfully reproduced the binding interface between Nap1 and an H2A/H2B dimer with a certain degree of accuracy. Here, we used a relatively low-resolution (∼6.7Å) crystal structure to validate the simulated structure. The broad peaks in the contact profile suggested that the binding between Nap1 and the H2A/H2B dimer is loose. Thus, using a low-resolution crystal structure to verify approximate binding sites in such a loose binding is reasonable.

### Nap1 C-terminal tails contribute to the binding to an H2A/H2B dimer in the simulations

**Figure 3.**
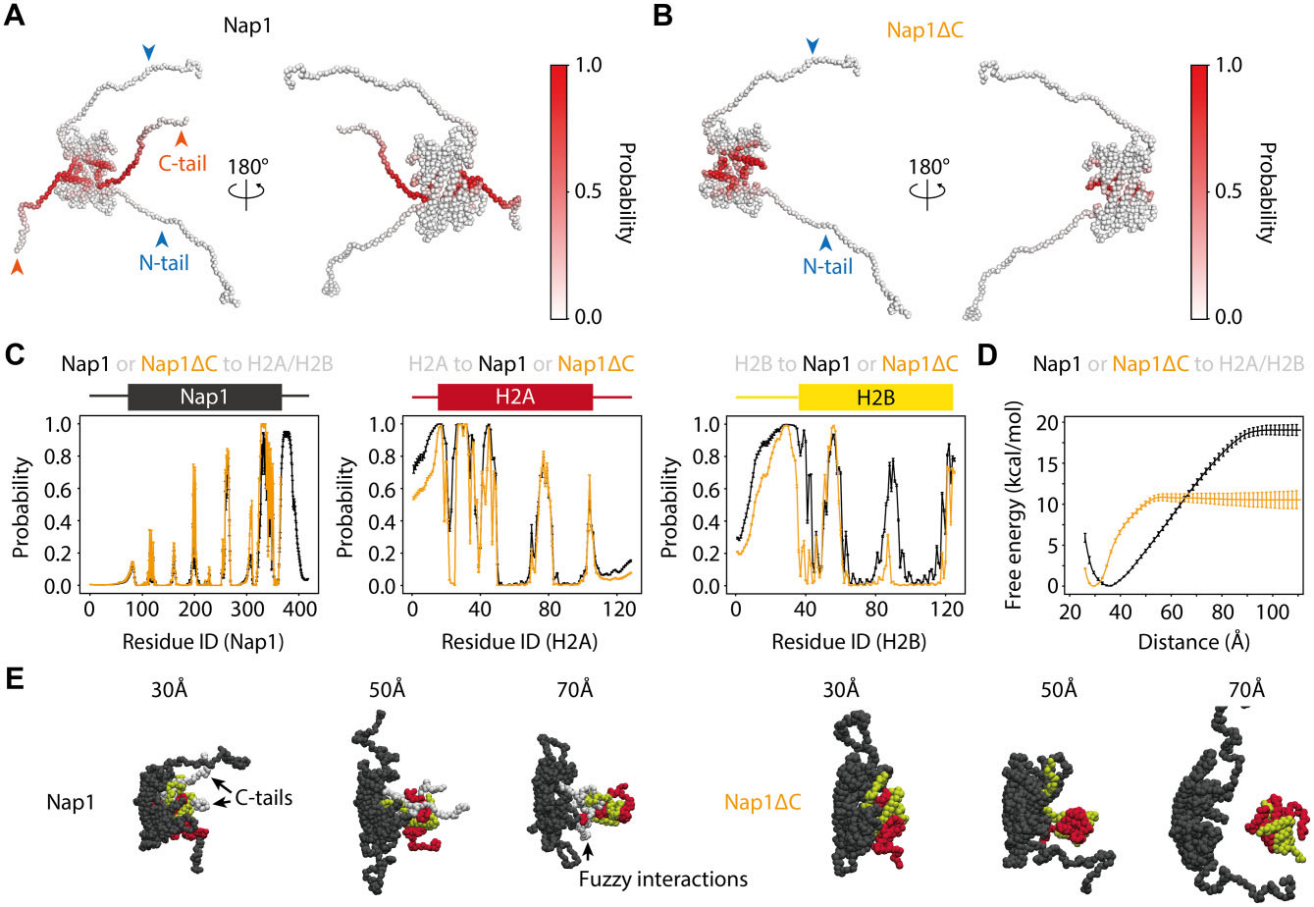
Coarse-grained molecular dynamics simulations of Nap1 or Nap1ΔC and an H2A/H2B dimer. (**A**) The structures of Nap1 in the one bead per one amino acid representation. The red gradient shows the probabilities of each residue contacting an H2A/H2B dimer in the simulations. (**B**) The structures of Nap1ΔC in the one bead per one amino acid representation. The color scheme is the same as in (A). (**C**) The probabilities of residues in Nap1 (Nap1ΔC) contacting H2A/H2B, in H2A contacting Nap1 (Nap1ΔC) and in H2B contacting Nap1 (Nap1ΔC). The black (yellow) line was calculated from the simulations of Nap1 (Nap1ΔC) and H2A/H2B. The error bars represent the SDs calculated from the 20 simulations. The bar/box cartoon above each plot represents flexible (bar) and globular (box) domains. (**D**) Free energy landscapes of Nap1 binding to H2A/H2B. The black (yellow) line was calculated from the umbrella sampling simulations of Nap1 (Nap1ΔC) and H2A/H2B. Error bars represent SDs calculated by randomly dividing datasets into three subdatasets. (**E**) The representative structures from the umbrella sampling simulations of Nap1 (left) or Nap1ΔC (right) and H2A/H2B. The reference distance for the harmonic potential is indicated above each structure. Nap1, H2A and H2B are colored gray, red and yellow, respectively.

One of the advantages of molecular dynamics simulations is their capability to model flexible tail dynamics. To investigate whether the tails are involved in the Nap1 binding to an H2A/H2B dimer, we performed 20 replicates of the simulations of full-length Nap1 or Nap1ΔC and a full-length H2A/H2B dimer for 1 × 10^8^ steps. The simulations showed that Nap1 C-terminal tails contacted an H2A/H2B dimer, but N-terminal tails did not (Figure 3A, C left), consistent with the previous experiment (8). On the other hand, the H2A N- (residues 1–15) and C-termninal tails (residues 106–128) and the H2B N-terminal tail (residues 1–36) contacted Nap1 (Figure 3C center, right). These results suggested that the Nap1 C-terminal tails are involved in Nap1 binding to an H2A/H2B dimer. Interestingly, Nap1ΔC hardly contacted a part of the H2B globular domain (residues 80–97) (Figure 3B, C right). Together, the Nap1 C-terminal tails provide additional interactions with the H2A/H2B dimer and stabilize the complex.

To quantitate the effects of Nap1 C-terminal tails on the binding, we calculated free energy curves by the umbrella sampling simulations of Nap1 with and without the C-terminal tails and an H2A/H2B dimer using the distance between the COM of Nap1 and H2A/H2B globular domains as a reaction coordinate (Figure 3D, E). We also calculated the differences between the minimum and plateau values of the curves. Then, we estimated standard binding free energy values (–19.3 kcal/mol and –12.6 kcal/mol for Nap1 with and without the C-terminal tails) by adding correction terms for the concentration and the constraint in the direction perpendicular to the reaction coordinates to the differences (38). The free energy value without the Nap1 C-terminal tail can be translated to a dissociation constant of 19 nM. The tight binding without the tail is consistent with previous reports (8). In the presence of the tails, the order of binding free energy (–19.3 kcal/mol) is consistent with that of the experimental value (–11.1 kcal/mol) (8). The slight discrepancy of the values may be attributed to (i) the ionic strength difference (when simulated at an ion concentration of 350 mM instead of 300 mM, which is the experimental condition, the binding free energies of Nap1 and Nap1ΔC are –12.2 kcal/mol and –7.3 kcal/mol, respectively), (ii) the inaccuracy of the potential energy functions and (iii) the inefficient sampling of the H2A/H2B dimer rotation. Besides those, the manner of binding (Figure 3E) may also contribute to the discrepancy as follows. First, the minimum free energy position shifted from 29.1 Å without tails to 35.3 Å with tails, suggesting the different manners of binding between Nap1 and Nap1ΔC. Second, the free energy value in the simulations increased as the COM distance increased from ∼38 to ∼100 Å. This gradual increase is attributed to the interactions via the Nap1 C-terminal tails (Figure 3E). In the previous experiment using fluorescence intensity changes as a measure, the fuzzy binding via the tails might be undetected because the diameter of the fluorescence quenching sphere is usually <20 Å (39). Therefore, the simulations indicated the role of the Nap1 C-terminal tails in the previously unappreciated fuzzy binding mechanism, although previous studies suggested that the C-terminal tails of human (40) and worm (41,42) Nap1 assist the binding to H2A/H2B. The quantitative contributions should be experimentally measured in the future.

### Nap1 induces H2A/H2B dismantling from a partially unwrapped nucleosome in the simulations

**Figure 4.**
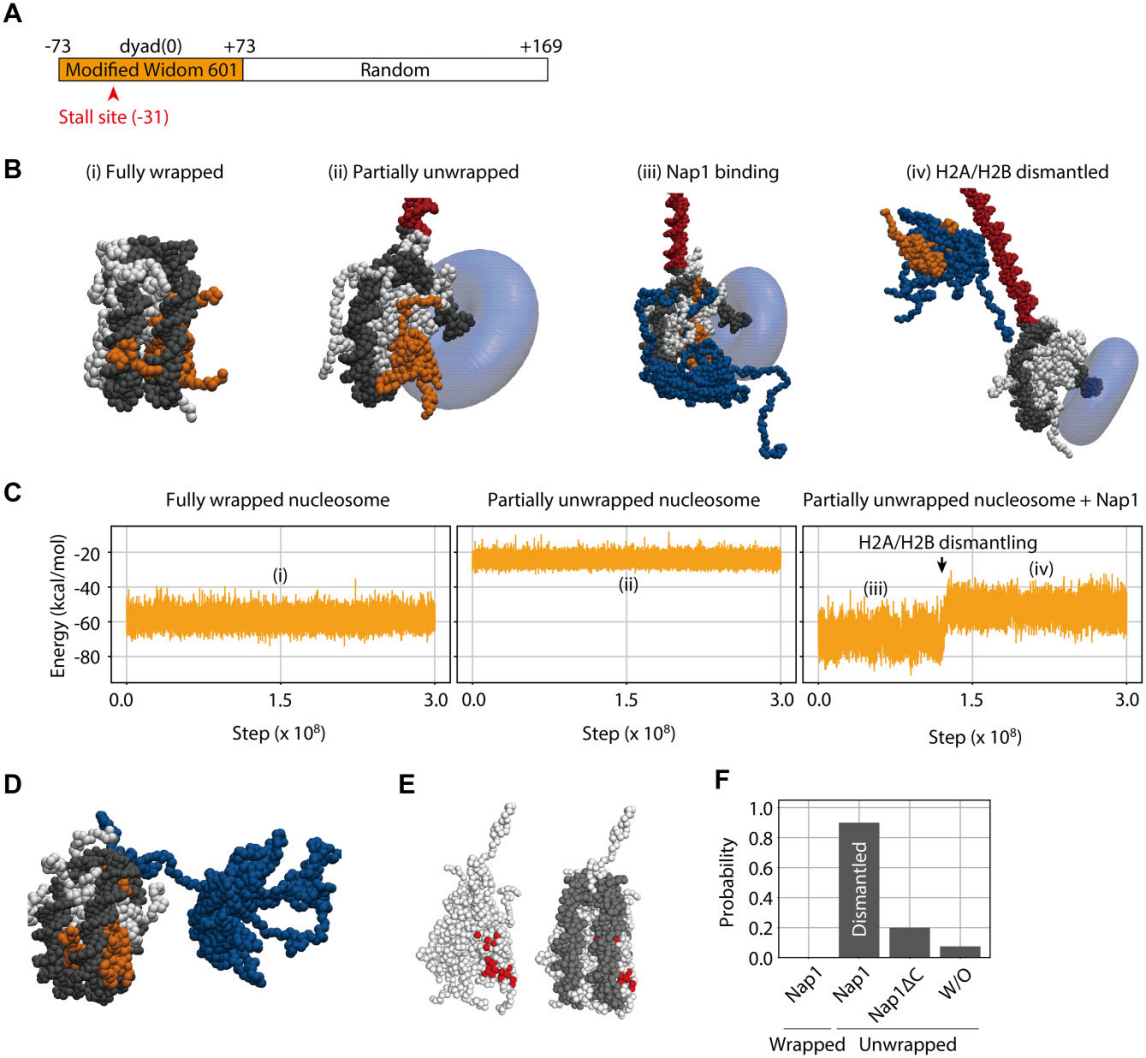
Coarse-grained molecular dynamics simulations of Nap1 and a nucleosome. (**A**) The DNA sequence in the simulations. (**B**) The representative snapshots from three sets of simulations where fully wrapped nucleosome (i), partially unwrapped nucleosome (ii) and partially unwrapped nucleosome bound to Nap1 (iii) and (iv) are initial structures. The translocase, the H2A/H2B dimer, the other histones and Nap1 are colored translucent blue, orange, white and blue, respectively. The nucleosomal DNA and downstream DNA are colored black and red, respectively. (**C**) The time course of the binding energy of H2A/H2B with the rest of the system in a representative trajectory where fully wrapped nucleosome (left), partially unwrapped nucleosome (center) and partially unwrapped nucleosome bound to Nap1 (right) are initial structures. (**D**) The representative snapshots from the simulations of Nap1 and a fully wrapped nucleosome. (**E**) The structures of a histone octamer and a nucleosome. The residues contacting Nap1 in the crystal structure (PDB ID: 5G2E) are colored red. (**F**) The probabilities of the H2A/H2B dismantling in the simulations. The first lane shows Nap1 with a fully wrapped nucleosome, the second and third lanes show Nap1 or Nap1ΔC with a partially unwrapped nucleosome and the fourth lane shows a partially unwrapped nucleosome without Nap1 (W/O).

Next, we performed the coarse-grained molecular dynamics simulations of Nap1 and a partially unwrapped nucleosome. First, the initial structure of the partially unwrapped nucleosome was prepared as described previously (4). Briefly, we modeled the translocase as a torus with an approximate size of a DnaB helicase. Then, the upstream linker DNA connected to a nucleosome [Figure 4B (i)] was threaded through the torus pore and pulled in a simulation so the nucleosome collided with the torus. After the collision, nucleosomal DNA began to unwrap. We used one of the simulation structures where the nucleosomal DNA was unwrapped up to the base pairs –31 bp from the dyad as the initial structure for the current simulations [Figure 4A, B (ii)]. Note that the torus and the base pair –31 bp from the dyad were anchored to the initial location so that partial unwrapping was maintained during the simulations.

In all the simulations of Nap1 and a partially unwrapped nucleosome (50/50), we observed that Nap1 bound to the H2A/H2B dimer exposed by the partial unwrapping [Figure 4B (iii); Supplementary Movie S1] and kept stably binding for 3 × 10^8^ steps, consistent with the tight binding of Nap1 to an H2A/H2B dimer. Interestingly, in 90% of the simulations (45/50), Nap1 dismantled an H2A/H2B dimer from a partially unwrapped nucleosome in 3 × 10^8^ steps [Figure 4B (iii), (iv), C (right), F; Supplementary Movie S1]. In these simulations, the downstream DNA (red in Figure 4B) sampled various orientations but did not associate with the histone core complex before or after the H2A/H2B dissociation. As a result of the simulations at a salt concentration of 350 mM, dismantling occurred in 54% of trajectories (27/50). This result showed that our simulation results are robust to changes in salt concentration to a certain degree. In contrast to these simulations, the dissociation of an H2A/H2B dimer from a fully wrapped and a partially unwrapped nucleosome was observed in 0 (0/80) and 7.5% (6/80) of the simulations in the absence of Nap1 [Figure 4B (i), (ii), C (left and center), F]. Strikingly, Nap1 did not bind to a fully wrapped nucleosome, and no H2A/H2B dismantling was observed (0/20) on the simulation time scale (Figure 4D, F; Supplementary Movie S2). As previously suggested (16,37), the binding interface between Nap1 and an H2A/H2B dimer in solution overlaps with that between DNA and an H2A/H2B dimer in a nucleosome and thus is precluded for the binding (Figure 4E). A previous study showed that overnight incubation at a low temperature (4°C) allows Nap1 to dismantle H2A/H2B dimers from a nucleosome (10). In this study, simulations at room temperature for a short time (approximately milliseconds) showed that H2A/H2B dismantling does not occur. These results suggested that the dismantling reaction from a fully wrapped nucleosome is energetically unfavorable compared with that from a partially unwrapped nucleosome. Together, the coarse-grained molecular dynamics simulations supported the experimental observation in this study that Nap1 induces H2A/H2B dismantling from a partially unwrapped nucleosome.

To obtain insight into the molecular mechanism by which Nap1 induces H2A/H2B dismantling from a partially unwrapped nucleosome, we calculated the average potential energy values of interactions between the H2A/H2B dimer and the other components in the system (Figure 4C). In the fully wrapped nucleosome, the H2A/H2B dimer interacted with DNA, the other H2A/H2B dimer and the H3/H4 tetramer, and the potential energy value was –58.3 ± 4.1 kcal/mol (Figure 4C left). When the translocase unwrapped the nucleosomal DNA up to –31 bp from the dyad, the value increased to –24.3 ± 2.5 kcal/mol (Figure 4C center). The Nap1 binding to the H2A/H2B dimer reduced the value to –67.9 ± 6.3 kcal/mol, and the H2A/H2B dismantling increased the value to –52.8 ± 5.3 kcal/mol (Figure 4C right). Therefore, an H2A/H2B dimer can gain more interaction energy when binding to Nap1 in solution (–52.8 ± 5.3 kcal/mol) than when binding in a partially unwrapped nucleosome (–24.3 ± 2.5 kcal/mol), which provides the driving force for H2A/H2B dismantling. Interestingly, there was an energetically favorable (–67.9 ± 6.3 kcal/mol) intermediate state where Nap1 binds to a partially unwrapped nucleosome. This state might also be the intermediate state for the nucleosome assembly in the absence of the translocase, which stabilizes the partially unwrapped nucleosome, consistent with previous observations (43,44). These thermodynamic considerations rationalized that Nap1 induces nucleosome assembly and H2A/H2B dismantling in the absence and presence of partial unwrapping, respectively.

### Roles of Nap1 C-terminal tails in H2A/H2B dismantling

In the EMSAs, we showed that Nap1ΔC hardly induces H2A/H2B dismantling from a partially unwrapped nucleosome (Figure 4B, C). To reveal the role of the C-terminal tails in the dismantling, we performed the coarse-grained molecular dynamics simulations of Nap1ΔC and a partially unwrapped nucleosome. In all the simulations (50/50), we observed Nap1ΔC bound to an H2A/H2B dimer exposed by partial unwrapping. However, Nap1ΔC kept binding throughout 3 × 10^8^ steps only in 62% of the simulations (31/50), consistent with the weaker binding of Nap1ΔC to an H2A/H2B dimer. Furthermore, in 20% of the simulations (10/50), we observed H2A/H2B dismantling (Figure 4F). Therefore, the simulation results also supported that the Nap1 C-terminal tails play a significant role in H2A/H2B dismantling from a partially unwrapped nucleosome.

**Figure 5.**
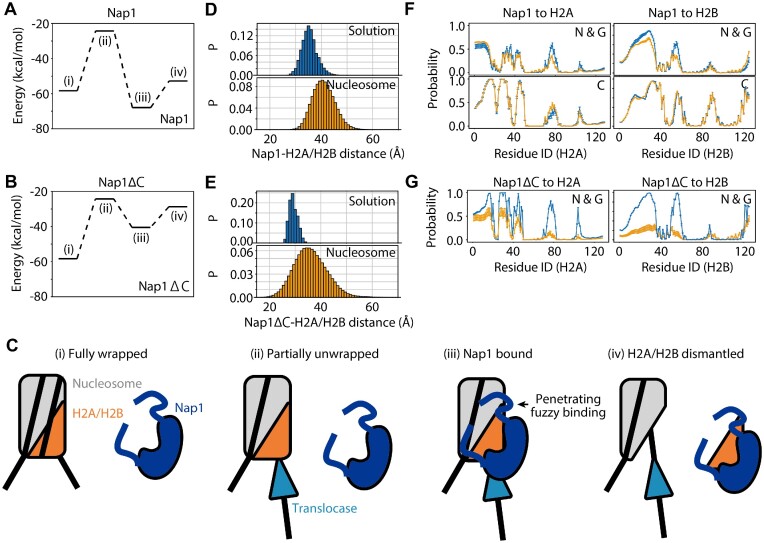
Comparison between Nap1 and Nap1ΔC. (A–C) The average potential energy values (**A, B**) and the cartoon **(C**) of each state when an H2A/H2B dimer binds to (i) a fully wrapped nucleosome, (ii) a partially unwrapped nucleosome, (iii) a partially unwrapped nucleosome bound by Nap1 (A) or Nap1ΔC (B), and (iv) H2A/H2B dimer bound by Nap1 (A) or Nap1ΔC (B). (**D, E**) The distributions of COM distance between an H2A/H2B dimer and Nap1 in (D) and Nap1ΔC in (E). The H2A/H2B dimer is in solution (top) or in a partially unwrapped nucleosome (bottom). (**F**) The probabilities of residues in Nap1 N-terminal and globular domains (top) or C-terminal domains (bottom) contacting H2A (left) or H2B (right) in solution (blue) or in a partially unwrapped nucleosome (yellow). (**G**) The probabilities of residues in Nap1ΔC N-terminal and globular domains contacting H2A (left) or H2B (right) in solution (blue) or in a nucleosome (yellow).

To gain insight into the role of the C-terminal tails in H2A/H2B dismantling, we again calculated the average potential energy values of interactions between the H2A/H2B dimer and the other components in the system (Figure 5A–C). As above, the energy values were –58.3 ± 4.1 and –24.3 ± 2.5 kcal/mol in fully wrapped and partially unwrapped nucleosomes, respectively. Nap1ΔC binding to an H2A/H2B dimer reduced the value to –40.5 ± 4.2 kcal/mol, and H2A/H2B dismantling increased the value to –28.7 ± 2.9 kcal/mol (Figure 5B). Therefore, the H2A/H2B dimer can gain marginally the same interaction energy when binding to Nap1ΔC in solution (–28.7 ± 2.9 kcal/mol) and when binding in a partially unwrapped nucleosome (–24.3 ± 2.5 kcal/mol), and thus is not efficiently dismantled. Interestingly, the energy loss upon the C-terminal tail truncation was more significant in the intermediate state where both the Nap1ΔC and the partially unwrapped nucleosome bound to the H2A/H2B dimer (–40.5 ± 4.2 kcal/mol) than in the product state where only the Nap1ΔC binds (–28.7 ± 2.9 kcal/mol). These results indicated that the Nap1 C-terminal tails modulate the manner of binding Nap1 to gain more interaction energy when an H2A/H2B dimer binds in a partially unwrapped nucleosome.

The distributions of COM distance between the globular domains of Nap1 and the H2A/H2B dimer supported that the binding conformation of Nap1 to an H2A/H2B dimer in a partially unwrapped nucleosome was altered by Nap1 C-terminal tails (Figure 5D, E). The mean COM distance values when full-length Nap1 and Nap1ΔC bound to an H2A/H2B dimer in a partially unwrapped nucleosome were 40.6 ± 4.3 Å and 35.5 ± 6.2 Å, respectively. Neither reached the most favorable position in solution (35 and 29 Å, respectively), and they tended to be located at 5–6 Å away from it in a partially unwrapped nucleosome (Figure 5D, E upper panels). Here, the distribution of the distance between Nap1 and H2A/H2B in solution is wider than that of Nap1ΔC due to the above-mentioned fuzzy binding via the C-terminal flexible tails. Accordingly, Nap1 tends to stay around the region where it can interact with H2A/H2B via the fuzzy binding, and the peak distance (40.6 ± 4.3 Å) of the distribution in a partially unwrapped nucleosome has a moderate probability in solution (0.03; 0.9 kcal/mol when translated to a free energy value) (Figure 3D). On the other hand, Nap1ΔC cannot bind in a fuzzy way, and the peak distance (35.5 ± 6.2 Å) of the distribution in a partially unwrapped nucleosome has a miniscule probability in solution (4.6 × 10^−4^; 3.4 kcal/mol). Therefore, the simulation results suggested that the fuzzy binding via the flexible tails plays a pivotal role in the H2A/H2B dismantling.

To further investigate the contribution of the Nap1 C-terminal tails to the Nap1 binding to an H2A/H2B dimer in a partially unwrapped nucleosome, we calculated the probabilities of each residue in H2A and H2B in a partially unwrapped nucleosome contacting residues in the Nap1 N-terminal tails or the globular domains from the simulations with Nap1 and Nap1ΔC (Figure 5F top, G yellow lines). As a control, we also calculated the probabilities from the simulations in which Nap1 or Nap1ΔC binds to an H2A/H2B in solution (blue lines). The comparison showed that the formation of a histone core complex by an H2A/H2B dimer hinders the Nap1 N-terminal tail and the globular domain binding to some parts of the H2A and H2B domains (H2A residues 73–82; H2B residues 1–36 and 49–60) for either Nap1 or Nap1ΔC. Notably, the H2A residues 73–82 and the H2B residues 49–60 collectively form the single structural domain partially buried in the interface between an H2A/H2B dimer and an H3/H4 tetramer, hence explaining the binding hindrance.

We also calculated the probabilities of each residue in H2A and H2B in the partially unwrapped nucleosome contacting residues in the Nap1 C-terminal tails from the simulation with Nap1 (Figure 5F bottom) and compared them with those from the simulations in which Nap1 binds to an H2A/H2B dimer in solution. Interestingly, the H2A/H2B dimer contact probabilities of Nap1 C-terminal tails were not significantly altered regardless of whether the H2A/H2B dimer was in a nucleosome or in solution. In particular, the buried structural domain mentioned above (H2A residues 73–82; H2B residues 49–60) was intimately contacted by the C-terminal tails. Together, the simulation results suggested that highly acidic C-terminal flexible tails of Nap1 contribute to the binding by associating with the binding interface buried and not accessible to the Nap1 globular domains.

## DISCUSSION

In this study, we performed *in vitro* transcription assays with the nucleosome substrates in the presence of a model chaperone, Nap1, and molecular dynamics simulations, revealing that Nap1 promotes H2A/H2B dismantling from a partially unwrapped nucleosome (Figure 5C). Also, the experiments and the simulations consistently supported that the Nap1 C-terminal tails play a significant role in the dismantling. The simulation results further suggested that the C-terminal tails contribute to the binding by associating with the binding interface buried and not accessible to the Nap1 globular domain. In the fuzzy binding of Nap1 C-terminal tails and the histone core complex, the flexible tails do not form a stable conformation even after the binding. This property would promote these tails accessing binding sites buried in the interface between H3/H4 and H2A/H2B. If a stable conformation is to be formed, steric hindrance between the tails and histones will prevent the binding.

The previous study showed that Nap1 removes an H2A/H2B dimer from a fully wrapped DNA or exchanges it with an H2A.Z/H2B dimer after 10 h of incubation at 4°C (10). The submillisecond time scale of the current simulations could not reproduce this hour time scale phenomenon. However, the simulations successfully reproduced H2A/H2B dismantling from a partially unwrapped nucleosome, suggesting that partial unwrapping by polymerases can dramatically reduce the time scale of H2A/H2B dismantling by Nap1. A previous study also showed that Nap1 promotes H2A/H2B dismantling from a nucleosome reconstituted on positively supercoiled circular DNA (11). Here, we used linearized DNA substrates and showed that Nap1 induces the H2A/H2B dismantling from a partially unwrapped nucleosome. In physiological conditions where a translocase such as RNAPs unwinds DNA and applies positive coiling stress on DNA in front (11), the positive DNA supercoiling and the DNA unwrapping may synergistically assist Nap1 to dismantle H2A/H2B dimers. This possibility should be addressed in the future.

In the current study, molecular dynamics simulations with a simple coarse-grained model showed that plenty of acidic residues in a disordered tail are the only requirement for penetrating fuzzy binding. On the other hand, the previous single-molecule magnetic and optical tweezer assays revealed that the histone chaperones FACT, SET/TAF Iβ and NPM induce dismantling of H2A/H2B dimers in the presence of DNA tension, which may partially unwrap nucleosomal DNA to some extent (45,46). Interestingly, these proteins commonly contain a disordered tail with many acidic residues. For example, residues 929–971 of the SPT16 subunit in the human FACT complex are in the intrinsically disordered C-terminal tail, and 55% are acidic residues (glutamate or aspartate) (47). In the case of the FACT complex, the previous study showed that the tails are required for FACT to dismantle H2A/H2B dimers from a destabilized nucleosome (48,49), as shown for Nap1 (72% of residues from 366 to 417 in the tail are acidic residues) in the current study. Further studies are necessary to confirm if the penetrating fuzzy binding mechanism applies to the cases of FACT, SET/TAF Iβ, NPM and other chaperones.

Our previous *in vitro* studies suggested that the collision between a model translocase, T7 RNAP, and a nucleosome induces downstream nucleosome repositioning by the lane-switch mechanism (4). In that study, we also showed that repositioning occurs after nucleosomal DNA is unwrapped up to –18 bp away from the dyad. The current study showed that a histone chaperone Nap1 dismantles an H2A/H2B dimer before nucleosomal DNA is unwrapped up to –31 bp away from the dyad. Therefore, the dismantling may precede and prevent downstream repositioning. While T7 RNAP, which we used in the current study, is believed not to specifically interact with a nucleosome, a eukaryotic translocase can possibly accomplish the (temporal) H2A/H2B dismantling via its own specific interactions with histones as the translocase proceeds through a nucleosome. Indeed, recent studies using cryogenic electron microscopy revealed that a collision between a eukaryotic RNA polymerase II and a nucleosome causes the loss of an H2A/H2B dimer (13,14). This prevention of downstream repositioning may underlie the molecular mechanism of maintaining the nucleosome positioning in transcription, histone recycling and nucleosomal DNA repair.

In a previous study, a spooling mechanism was experimentally proposed in which the upstream DNA reassociation with the region on the histone core complex exposed by unwrapping leads to repositioning of the histone core complex to the upstream DNA (50). In the current study, we truncated upstream DNA to reduce computational cost and focus on H2A/H2B dissociation instead of repositioning. Therefore, it is impossible to reproduce the spooling mechanism by the current simulation setup. Also, detailed structural modeling of polymerase and bent DNA inside it may be required to reproduce the spooling mechanism. Simulations using such a model will be tried in the future.

In the current study, we performed coarse-grained molecular dynamics simulations to obtain insight into the structural dynamics of H2A/H2B dismantling from a partially unwrapped nucleosome by Nap1. Although the model parameters have been carefully tuned for the intranucleosome interactions (26), only electrostatic and excluded volume interactions were applied to the interactions between Nap1 and a nucleosome. This simple treatment boosted the calculation speed with compromised accuracy. The binding interface and affinity between Nap1 and H2A/H2B globular domains were reasonably well reproduced, although they can be improved by additionally considering hydrophobic interactions. Despite the margin for improvement, the molecular dynamics simulation technique is one of the powerful approaches to elucidating structural dynamics of flexible protein regions such as intrinsically disordered tails, as demonstrated here.

## DATA AVAILABILITY

The data that support the findings of this study are available from the corresponding author upon reasonable request. The input and trajectory files have been submitted to the Biological Structure Model Archive (BSM-Arc) under BSM-ID BSM00044 (https://bsma.pdbj.org/entry/44).

## Supplementary Material

gkad396_Supplemental_Files
