# Supplementary material for: Histone chaperone Nap1 dismantles an H2A/H2B dimer from a partially unwrapped nucleosome
Source: Nucleic Acids Res. 2023 May 13;51(11):5351–63. doi: 10.1093/nar/gkad396 (PMC10287947; doi:10.1093/nar/gkad396)
Supplement: gkad396_Supplemental_Files [file gkad396_supplemental_files.zip › Supplement.pdf]

Supplementary data for

Histone chaperone Nap1 dismantles an H2A/H2B dimer from a partially unwrapped nucleosome

Fritz Nagae[1], Shoji Takada[1], Tsuyoshi Terakawa[1,2,*]

[1]Department of Biophysics, Graduate School of Science, Kyoto University, Kyoto, Japan.

[2]PREST, Japan Science and Technology Agency (JST), Kawaguchi, Japan.

[*]To whom correspondence should be addressed: terakawa@biophys.kyoto-u.ac.jp

Table S1

The DNA sequence used in simulations or experiments. The modified 601 sequence, the T7 promoter sequence, and the DNA translocase stall sites are colored red, green, and blue, respectively.

Name	Used in	Sequence
Unwrapped_601_random	Simulations	AGCTCTAGCACCGCTTAAACGCACGTACGCGCTGTCCCCCGCGTTT<br>TAACCGCCAAGGGGATTACTCCCTAGTCTCCAGGCACGTGTCAGAT<br>ATATACATCCTGTTGCTAGCTCGGTGACGGTTCAATGTACGCTGTC<br>GGCCCTTCAGCGCTGGTGTGCCTGTTATGGACAAGCTATTGTGAAG<br>TGGTCTTCCAATTCGAT
T7_601_-14th	Experiments	TTTGCGCTCAGCCCATTTGTGGAAGATGCGTTCCCAATGACCTTCC<br>GCAGATAAGCGGGTAAAATCCCGACTATGGTCGCTTTTGTGCTCAC<br>GGCATTAATACGACTCACTATAGGGTTTTCGTTGTTTTTTTCTGTC<br>TCGTGCCTGGTGTCTTGGGTGTTTTCCCCTTGGCGGTTAAAACGCG<br>GGGGACAGCGCGTACGTGCGTTTAAGCGGTGCTAGAGCTGTCTACG<br>ACCAATTGAGCGGCCTCGGCACCGGGATTCTGATAGGTGGTTTGAG<br>TGCACAGTCGGGATTGGATAGAAAGGAGCCGTGTAGAATTCCTCTC<br>AGAGTCACTGCGCAGTAGGGGCAAGAATCTTTAGTAGGTCAGTCGG<br>CGTTGGGTTAGCATGCCGATTTCTAGTCAGTGGATACGCCAAATGC<br>CTTCATGCGCCCAAAACATGTTGGCATGTTCACGATGTGCCCAACG<br>ATGTCCTTCCCAACGTGCTCTACCGGCATGATCAAAGC
T7_601_-69th	Experiments	TTTGCGCTCAGCCCATTTGTGGAAGATGCGTTCCCAATGACCTTCC<br>GCAGATAAGCGGGTAAAATCCCGACTATGGTCGCTTTTGTGCTCAC<br>GGCATTAATACGACTCACTATAGGGTTTTCGTTGTTTTTTTCTGAC<br>TCGTGCCTGGTGTCTTGGGTGTTATCCCCTTGGCGGTTAAAACGCG<br>GGGGACAGCGCGTACGTGCGTTTAAGCGGTGCTAGAGCTGTCTACG<br>ACCAATTGAGCGGCCTCGGCACCGGGATTCTGATAGGTGGTGAATT<br>CTTGAGTGCACAGTCGGGATTGGATAGAAAGGAGCCGTGTACTCTC<br>AGAGTCACTGCGCAGTAGGGGCAAGAATCTTTAGTAGGTCAGTCGG<br>CGTTGGGTTAGCATGCCGATTTCTAGTCAGTGGATACGCCAAATGC<br>CTTCATGCGCCCAAAACATGTTGGCATGTTCACGATGTGCCCAACG<br>ATGTCCTTCCCAACGTGCTCTACCGGCATGATCAAAGC

FIGURES

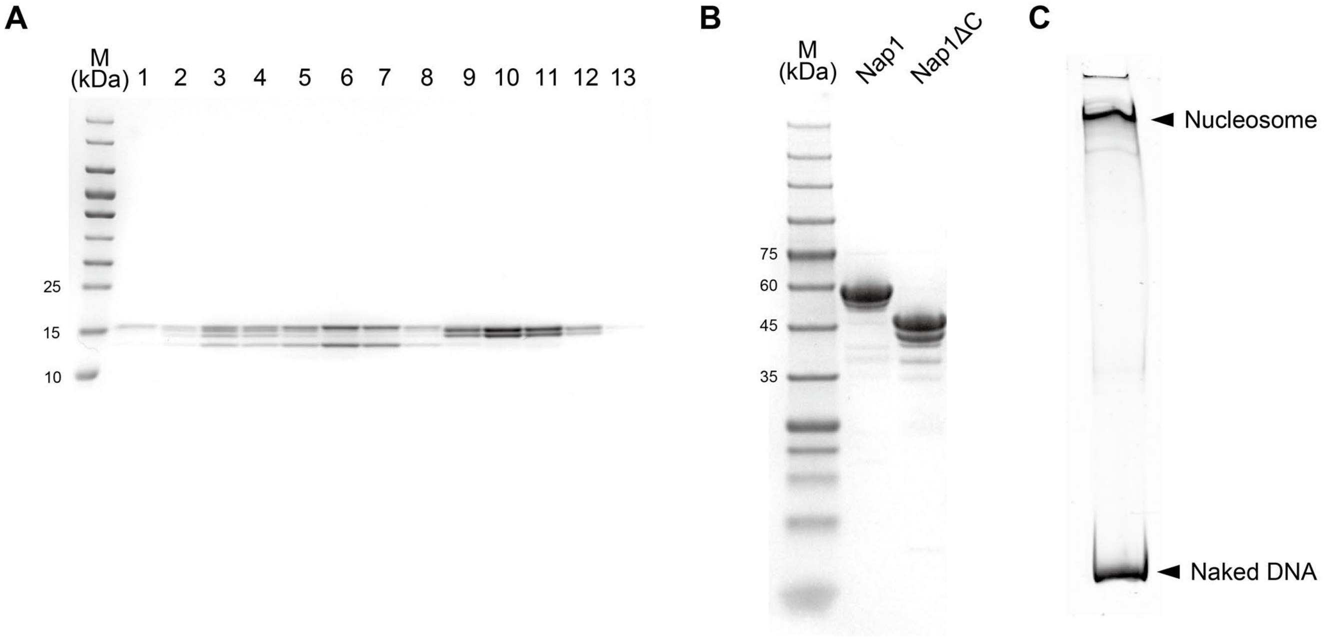

Figure S1

Nucleosome reconstitution and Nap1 purification. (A) A gel image of 5-20% SDS-PAGE of reconstituted histone octamers. The samples were run on the gel at 21 mA and 25°C for 75 minutes. The fractions 2 (F2), 3 (F3), and 4 (F4) were pooled and used to reconstitute nucleosomes. “M” stands for a marker (Thermo Fisher SCIENTIFIC; 26616) run for protein size estimation. (B) A 5-20% SDS-PAGE gel image of Nap1 and C-terminal-tail-truncated Nap1 (Nap1ΔC). The samples were run on the gel at 21 mA and 25°C for 75 minutes. “M” stands for a marker (ATTO; WSE-7020). (C) A gel image of 6% polyacrylamide gel electrophoresis of the reconstituted nucleosomes in a native condition. The sample was run on the gel at 10.5 mA and 4°C for 2.5 hours.

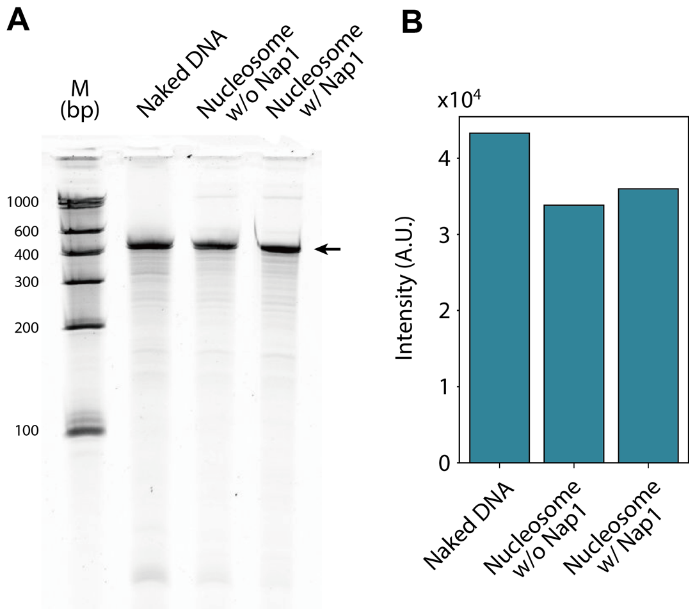

Figure S2

Transcription of naked DNA and DNA reconstituted with a nucleosome in the presence or absence of Nap1. (A) 15% Urea-TBE gel electrophoresis of transcription products in 0.5×TBE buffer. The samples were run on the gel at 21 mA and 25℃ for 75 minutes. “M” stands for a marker (Thermo Fisher SCIENTIFIC; SM1831). (B) The intensity of the bands pointed by the black arrow in (A).

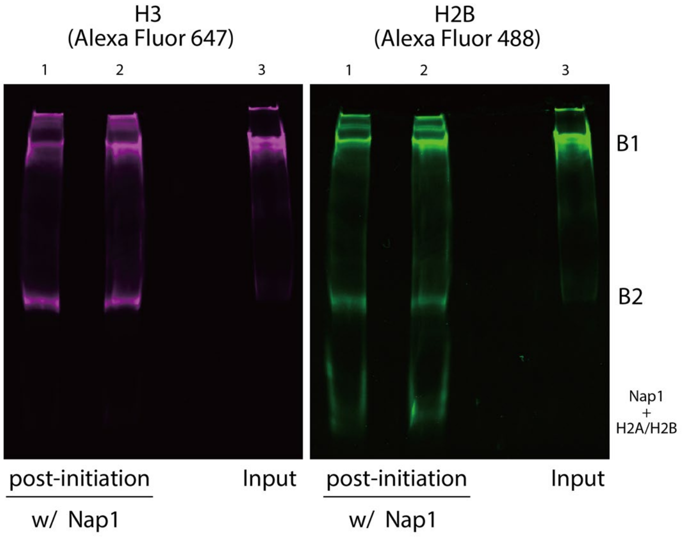

Figure S3

Native PAGE gel images of the reaction products using DNA reconstituted with a nucleosome in the presence of T7 RNAP (post-initiation of transcription) and the presence or absence of Nap1. The nucleosome was reconstituted with Alexa Fluor 647 labeled H3, Alexa Fluor 488 labeled H2B, H4, and H2A. The same gel was imaged using the filters for Alexa Fluor 647 (left) and Alexa Fluor 488 (right), respectively. Samples from two repeated experiments were run in lanes 1 and 2, showing reproducibility.

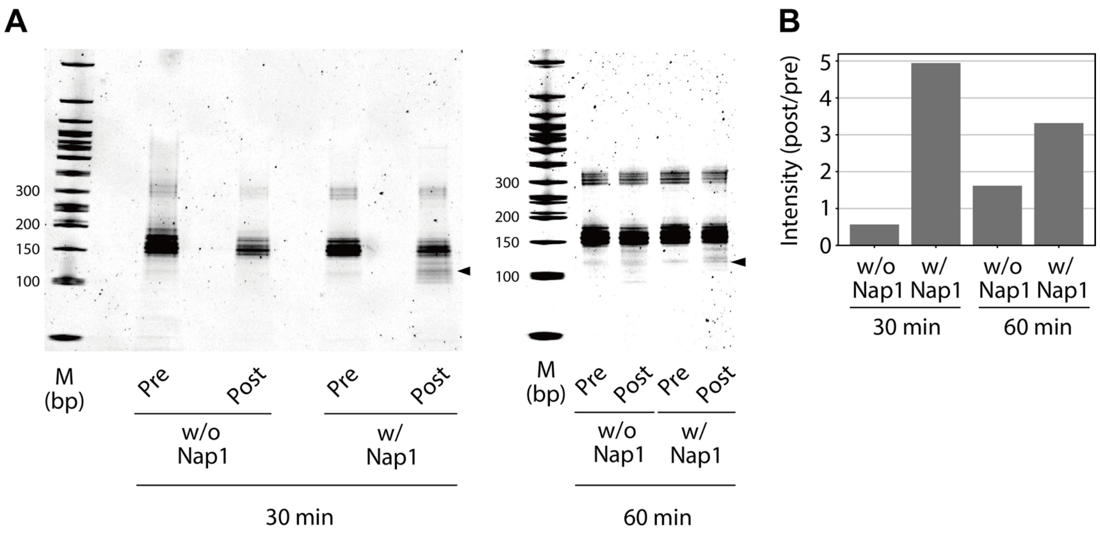

Figure S4

MNase assay of nucleosome/T7 RNAP collision products in the presence and absence of Nap1. (A) Gel images of 5-20% native PAGE of the products digested by MNase. The reaction products were incubated with MNase for 30 (left) and 60 (right) minutes, respectively. “Pre” and “Post” represent pre-transcription-initiation and post-transcription-initiation, respectively. The arrowheads point to the ~120 bp bands. “M” stands for a marker (N0556S; New England Biolabs). (B) The intensity of the ~120 bp band normalized by the pre-transcription-initiation values.

Movie S1 (Separate file)

A representative trajectory of H2A/H2B dismantling by Nap1 (dark blue) from the partially unwrapped nucleosome (an exposed H2A/H2B, orange; other histones, white; DNA, black).

Movie S2 (Separate file)

A representative trajectory of Nap1 diffusing around a fully wrapped nucleosome. The color scheme is the same as in Movie S1.
